# Supplementary material for: Taxonomic analysis of metagenomic data with kASA
Source: Nucleic Acids Res. 2021 Mar 30;49(12):e68. doi: 10.1093/nar/gkab200 (PMC8266618; doi:10.1093/nar/gkab200)
Supplement: gkab200_Supplemental_Files [file gkab200_supplemental_files.zip › Suppl_D.zip!/OPAL_comparison_new/gold_standard/rarefaction_curves_log_scale.pdf]

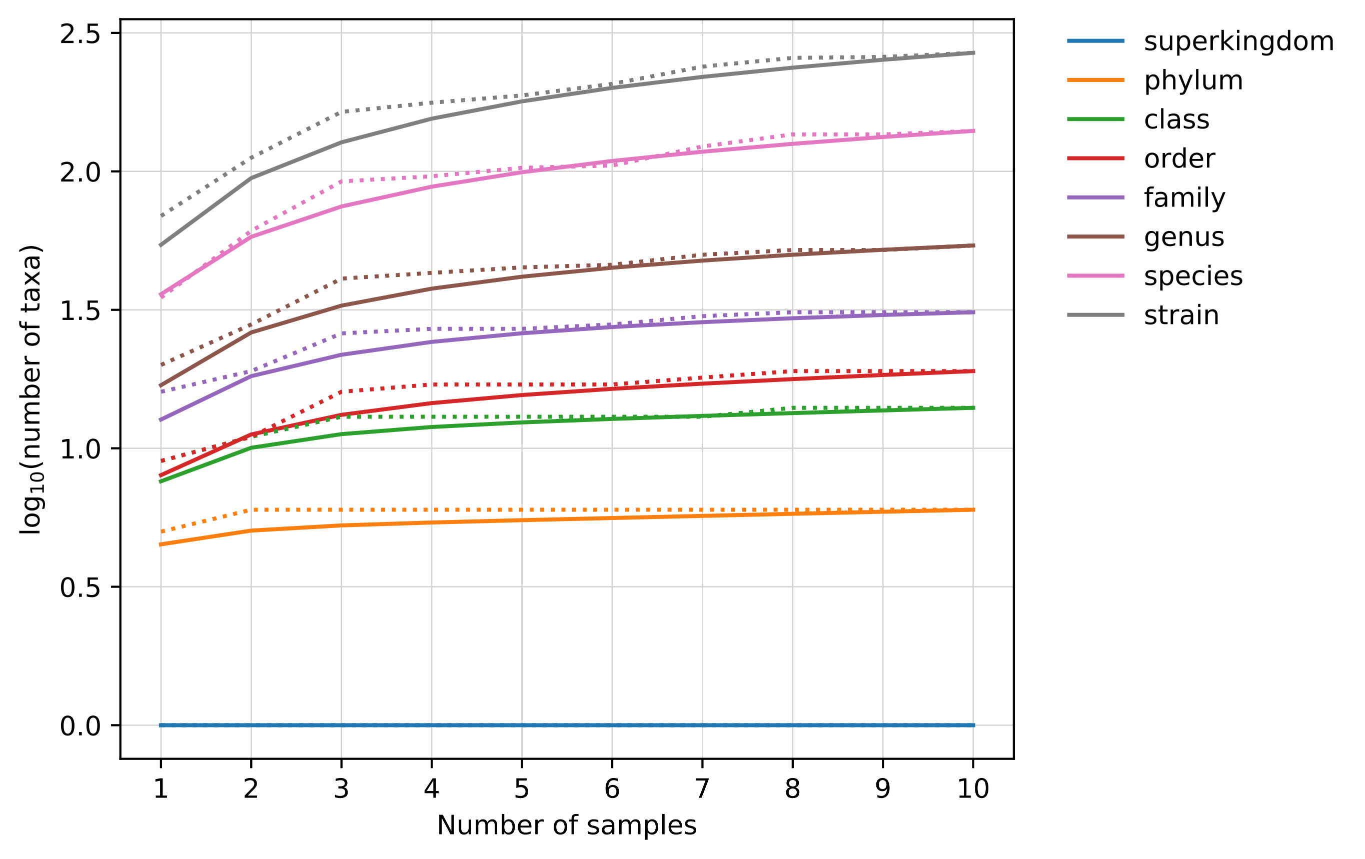

superkingdom
phylum
class
order
family
genus
species
strain
log10(number of taxa)
Number of samples
0.0
0.5
1.0
1.5
2.0
2.5
1
2
3
4
5
6
7
8
9
10
